# Multi-Objective Optimization of The Low-Pressure Casting of Large-Size Aluminum Alloy Wheels through a Systematic Optimization Idea

**DOI:** 10.3390/ma16186223

**Published:** 2023-09-15

**Authors:** Yuhang He, Dehong Lu, Zhenming Li, Donghui Lu

**Affiliations:** 1 Faculty of Materials and Science Engineering, Kunming University of Science and Technology, Kunming 650500, China; jackson.y.he@outlook.com; 2 Yunnan Fuyuan Jinfei Wheel Manufacturing Co., Ltd., Qujing 655000, China; lizhenming516@163.com (Z.L.); ldh19871025@163.com (D.L.)

**Keywords:** systematic optimization, large-size aluminum alloy wheel, low-pressure casting, numerical simulation, RSM, NSGA-II

## Abstract

The process parameters in the low-pressure casting of large-size aluminum alloy wheels are systematically optimized in this work using numerical casting simulation, response surface methodology (RSM), and genetic algorithm (NSGA-II). A nonlinear input–output relationship was established based on the Box–Behnken experimental design (BBD) for the crucial casting parameters (pouring temperature, mold temperature, holding pressure, holding time), and response indicators (defect volume fraction, spokes large plane mean secondary dendrite spacing (SDAS)), and a mathematical model was developed by regression analysis. The Isight 2017 Design Gateway and NSGA-II algorithm were used to increase the population and look for the best overall solution for the casting parameters. The significance and predictive power of the model were assessed using ANOVA. Casting numerical simulation was used to confirm the best option. To accomplish systematic optimization in its low-pressure casting process, the mold cooling process parameters were adjusted following the local solidification rate. The results showed that the mathematical model was reliable. The optimal solutions were a pouring temperature of 703 °C, mold temperature of 409 °C, holding pressure of 1086 mb, and holding time of 249 s. The mold cooling process was further optimized, and the sequence solidification of the optimal solution was realized under the optimized cooling process. Finally, the wheel hub was manufactured on a trial basis. The X-ray detection, mechanical property analysis, and metallographic observation showed that the wheel hub had no X-ray defects and its mechanical properties were well strengthened. The effectiveness of the system optimization process scheme was verified.

## 1. Introduction

Currently, lightweight technology is one of the most efficient ways to reduce emissions and save energy. It is used in a variety of industries, including the aerospace, automotive, and military industries [1]. Aluminum alloy is a lightweight material that is commonly utilized in the manufacture of automobiles due to its corrosion resistance, lightweight, and good thermal conductivity [2]. According to several research reports, switching from steel to aluminum wheels can lower their bulk by up to 50%. Due to the outstanding weight reduction and fuel efficiency of aluminum alloy, the fuel consumption is accordingly lowered by 6–8% for every 10% of weight reduction in the vehicles [3,4]. China, however, is still in the early stages of replacing large-size steel wheels; “Large size” wheels are mainly used in heavy-duty vehicles and buses and other large vehicles, which have a larger spoke surface and need to have a stronger load capacity. The manufacturing process now faces greater demands and difficulties in attaining lightweight components while also guaranteeing the durability of the wheels. Cast aluminum alloys are favored in the automotive manufacturing industry due to economic considerations, and low-pressure casting is currently the most common method for producing large-size aluminum alloy wheels [5].

The advantages of low-pressure casting include smooth filling, high production efficiency, and good product quality [6,7]. However, the application of low-pressure casting, particularly for large-sized wheels, can frequently result in defects and reduced properties if not applied correctly. The load demanded by large bus wheels, for instance, results in mechanical property requirements that are higher than those of normal-size wheels. Additionally, due to the significant variation in wall thickness at their load-bearing structure and the size of their spoke surfaces, they are difficult to solidify sequentially. The control of these issues’ emergence is closely correlated with the process parameters for low pressure. The conventional method of changing one process parameter at a time, which does not fully account for the interaction between factors and cannot achieve systematic optimization, is often used to examine the impact of process parameters on casting defects and performance [8].

To optimize the die-casting process parameters, several clever optimization algorithms and experimental techniques have recently been developed. Zhang et al. [9] have reported a neural network model combining a quantitative learning vector and back propagation (BP) algorithm to map the LPDC process parameters and complex relationships between evaluation metrics. They also proposed a strategy combining artificial neural networks (ANN) and genetic algorithms (GA) to optimize the LPDC process. To improve the die-casting process parameters for aluminum alloy carburetor shells, Arun Kumar et al. [10] coupled the GA method and fuzzy logic method (FLM). They then utilized analysis of variance (ANOVA) to assess the significant level of each process parameter’s contribution to the percentage of defects. An E-TKNN method has been developed by Deng et al. [11] to find novel die-casting process parameters. Experiments have confirmed the method’s viability and superiority, and the authors have also proposed a new idea for designing additional casting process parameters. Wang et al. [12] adopted the Taguchi method to optimize the process variables for ZL205A cylindrical shell alloy parts made by low-pressure casting, and the porosity values of the castings were significantly decreased. To analyze casting defects, A. Kumaravadivel et al. [13] combined the Six Sigma method with the response surface methodology (RSM) to the flywheel casting process in the foundry to minimize the defects in this process. The results showed that by using such a model, one can obtain remarkable savings in time and cost. RSM was utilized by Li et al. [14] to develop a numerical simulation strategy for the efficient elimination of casting flaws to identify the ideal set of process parameters. To evaluate the relevance and predictive potential of the mathematical model created using regression analysis, a total of 17 simulations based on Box–Behnken and ANOVA were created.

Designers may now numerically simulate the casting process and anticipate casting quality, defect distribution, etc., thanks to the development of casting CAE, enabling multi-factor analysis and quantitative evaluation of casting quality [15,16,17]. To improve the simulation’s accuracy, A U Saadah et al. [18] used Abaqus to numerically simulate the heat transfer coefficient (HTC) of low-pressure cast wheels made of aluminum alloys. They then compared the simulation results with experimental results to determine the HTC as a function of the initial mold temperature. To compare the variations in temperature fields through numerical simulations and experiments to establish a numerical model of temperature to correct the simulated temperature fields, Zheng et al. [19] studied the solidification behavior of aluminum alloy wheels during low-pressure casting. The effects of wheel microstructure and flaws on impact performance under LPDC were investigated and analyzed by Mattia Merlin [20] using numerical simulations. To simulate low-pressure cast A356 aluminum alloy wheels, P. Fan et al. [21] developed a thermal-fluid-composition model based on Fluent. This model successfully predicted the pore size distribution and pore number density. To model the low-pressure casting process, Lin et al. [22] employed the ProCAST Visual-Cast 13.5 software to simulate the temperature field of an aluminum alloy. A process design to successfully reduce shrinkage was proposed after a thorough analysis of the changes in the temperature field, velocity field, solid phase rate, and defect location was carried out using the ProCAST software. By numerically modeling the solidification period at various phases of a part, Sui et al. [23] were able to establish the optimum cooling and holding process for an automobile wheel, which significantly decreased the defect rate.

At present, there is limited research on the optimization of process parameters and the exploration of new solidification control methods in the low-pressure casting process of large-size aluminum alloy wheels, and most ignore the coupling effect between the process parameters, and further studies are needed in the systematic optimization. As a result, the large-size aluminum alloy wheel used in this study serves as the research topic. The experiment was designed using the RSM response surface, and a commercial process simulation software, ProCAST, was used to simulate the casting process. The response results and test parameters were then fitted to create the prediction model, and ANOVA was used to assess the model’s accuracy. The regression model was then optimized for several objectives using the NSGA-II genetic algorithm. After the best option was found, the cooling process parameters were adjusted to assure sequential solidification and achieve systematic optimization to the fullest.

## 2. Materials and Methods

**Figure 1:**
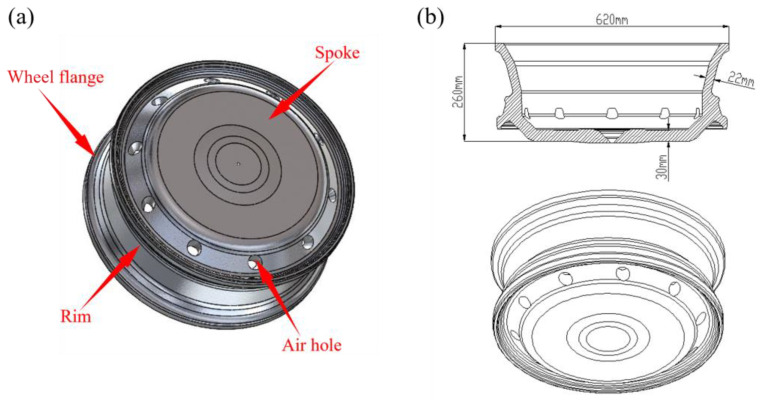
Schematic diagram of the wheel: (**a**) Schematic diagram of wheel casting, (**b**) CAD dimensions of the wheel.

**Figure 2:**
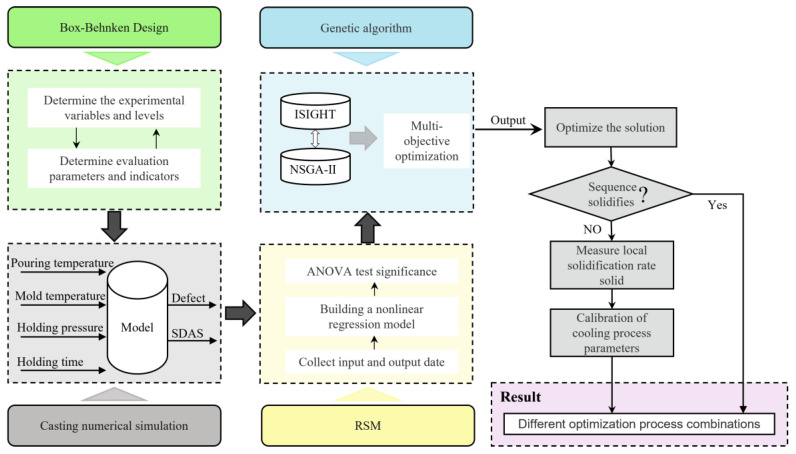
Experimental flow chart.

The wheel castings and dimensions are presented in Figure 1, and the A356 aluminum alloy, whose chemical composition is described in Table 1, was chosen as the wheel material. In this work, we simulate the low-pressure casting of the wheel based on response surface design using the commercial process simulation software, taking into account the impact of various process factors on the overall performance of the wheel. Specifically, it emphasizes holding pressure, holding time, mold temperature, and pouring temperature optimization. The specific process and steps are shown in Figure 2.

### 2.1. Optimized Experimental Design

#### 2.1.1. Determination of Test Variables

The pouring temperature is one of several factors that affects low-pressure casting. The front end of the metal liquid may solidify before the filling process is complete if the pouring temperature is too low, resulting in cold shuts, misruns, and underfilling. When the temperature is too high, the metal liquid is more likely to absorb hydrogen gas from the atmosphere increasing the tendency for H2-related porosity, and the mold is subjected to more severe thermal erosion and thermal cracking, which shortens its useful life [24]. Therefore, combined with actual production experience, the selection range of control casting temperature is between 690 and 710 °C.

The mold temperature has a direct impact on the casting quality; a lower mold temperature will cause the metal liquid to flow poorly, resulting in underfilling [25], whereas a higher mold temperature will slow the rate at which the metal solidifies, giving the casting a courser microstructure and increasing shrinkage and porosity. At present, in most of the wheel hub manufacturing, the mold temperature is mostly controlled between 350–500 °C, so in this paper, combined with production experience, the mold temperature selection range is 350–450 °C.

With the increase in the holding pressure, this will speed up the heat transfer between the casting and the mold during the solidification process, shorten the solidification time, and make the structure denser, but too high holding pressure will also affect the surface roughness and dimensional accuracy of the casting, so it is necessary to set the holding pressure reasonably [26]. Based on Huang et al. [27]’s idea of reducing hub defects by pressurizing the hub locally, this paper selects a large range of holding pressures to explore the influence of holding pressure on hub castings. Therefore, the selection range is between 700 and 1200 mb.

The holding time is another crucial variable in the holding phase in addition to the holding pressure. If the holding period is insufficient, the casting’s solidification will not be fully supplied, leading to rapid shrinkage and the formation of shrinkage holes as well as the phenomena of reflux at the casting’s bottom because it is not solidified. If the holding period is too lengthy, it will not only degrade production efficiency but also cause the lower end of the gate to solidify, making it difficult to release the mold [28]. Dong et al. [29] studied the pressure holding time of aluminum alloy wheels, and the selection range was controlled within 140–160 s. In view of the fact that the research object is a larger hub, the required pressure holding time is also increased, and to find the optimal solution in a larger range, the pressure holding time is set between 220–260 s.

As a result, the test variables of pouring temperature, mold temperature, holding pressure, and holding period were chosen, as shown in Table 2.

#### 2.1.2. Generate BBD Experimental Protocols

The Box–Behnken (BBD) experimental design was utilized after deciding on the test variables and levels, as indicated in Table 3. The BBD experimental design is an experimental design method that can evaluate the nonlinear relationship between indicators and factors. The BBD test is different from the central repeat test in that it does not require multiple tests in succession, and under the same factors, the test combination is less than the central repeat test and therefore more economical [30]. When variable factors and levels are entered into the BBD Design-Expert 13 software, it can automatically generate test tables. As can be seen from the table, a total of twenty-nine groups of test schemes were generated according to BBD, including five groups of central repeated tests.

#### 2.1.3. Determination of Assessment Parameters and Indicators

The majority of the current evaluations of their casting quality are based on the defect volume fraction since shrinkage is the most prevalent flaw in the low-pressure casting of large-size aluminum alloy wheels [31]. In this paper, the Niyama criterion is used to quantitatively predict the defect rate of large-size aluminum alloy wheel casting. The criterion of the mathematical model of [32]:

(1) $$Ny^{*}=\frac{G\lambda_{2}\sqrt{∆P_{cr}}}{\sqrt{\mu \beta ∆T_{f}R}}$$

where Ny* is the value of the criterion function; *G* is the temperature gradient, °C/m; *λ*_2_ is the secondary dendrite spacing, m; Δ*P_cr_* is critical depressurization, Pa; *μ* is the dynamic viscosity of the alloy, Pa·s; *β* is the total solidification shrinkage; Δ*T_f_* is the crystallization temperature range, °C; *R* is the cooling rate, °C/s.

**Figure 3:**
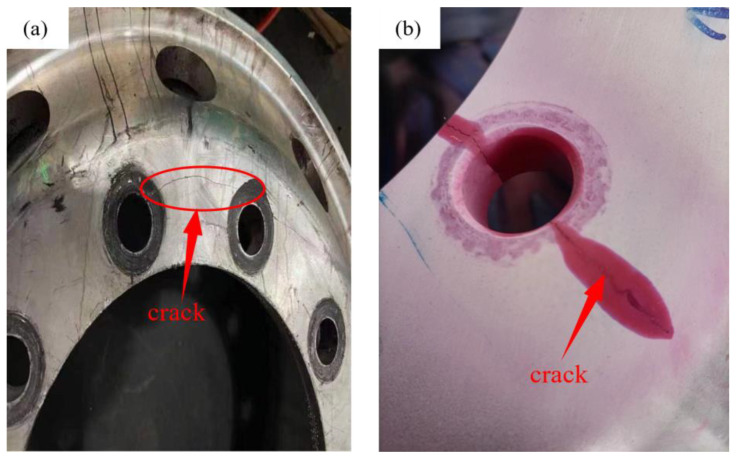
Cracks location map: (**a**) Hub crack, (**b**) Crack development diagram.

Additionally, the secondary dendrite spacing (SDAS) has a significant impact on the casting’s mechanical attributes. As shown in Figure 3, most of the hub cracks occur near the bolt holes on the large flat surface of the spoke, which is a stress-concentrated area. SDAS can effectively reflect the mechanical properties of castings. The smaller the SDAS is, the denser the structure of castings and the better their mechanical properties. Thus, effectively reducing the secondary dendrite spacing here is essential. Therefore, it should be clear that the optimization standard of mechanical properties at the large plane of the wheel spoke should be improved as much as possible under the premise of no X-ray grade defects in the casting.

In this paper, the Fisher–Kurz model is used to calculate the secondary dendrite spacing [33]:

(2) λ2=KM·tf13

where *λ_2_* is the secondary dendrite spacing; *t_f_* is the solidification time of a certain spatial position, sec; *M* is the alloy property constant, microns 3/s; *K* is the correction factor. For A356 aluminum alloy, the *M* rough value is 680 microns 3/s. The correction coefficient *K* can be obtained by mathematical approximation according to the test data of secondary dendrite spacing. When *K* is 0.89, the numerical consistency between simulated SDAS and measured SDAS is higher.

### 2.2. Simulation Based on Response Surface Design

#### 2.2.1. Build the Model

**Figure 4:**
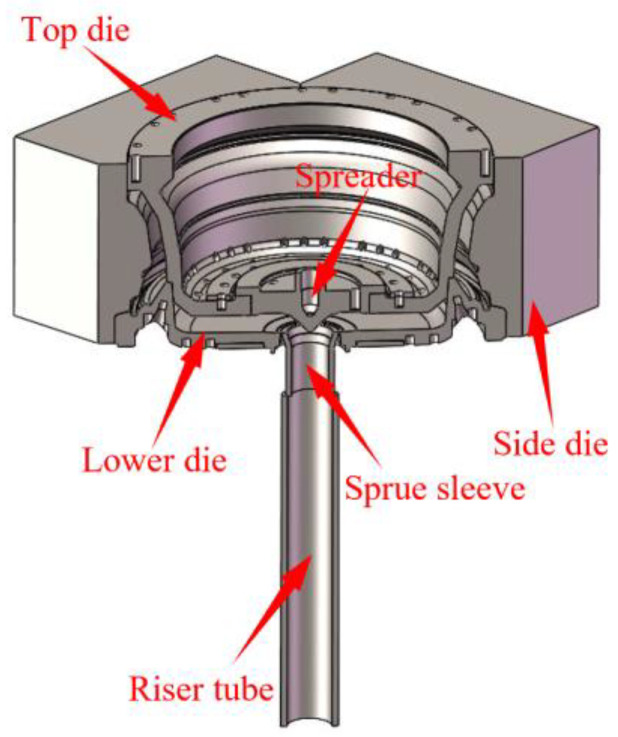
Three-dimensional cross-sectional view of the wheel mold cooling system of the mold.

**Figure 5:**
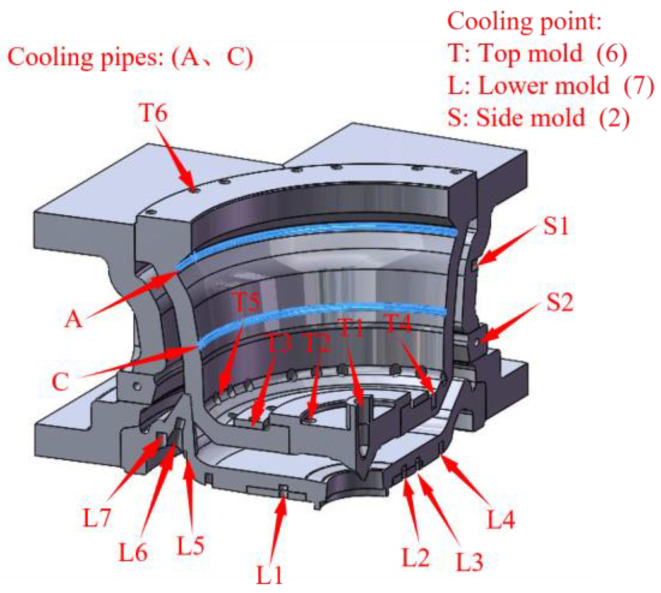
Schematic diagram of the overall.

**Figure 6:**
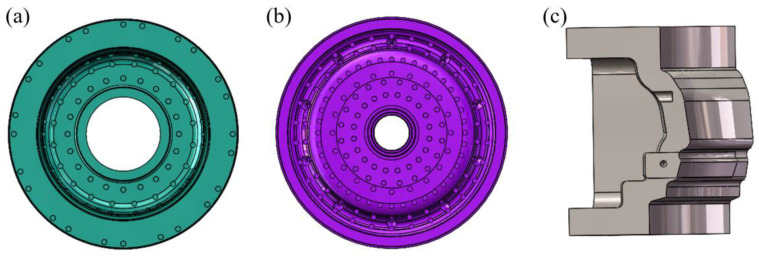
Schematic diagram of each mold cooling: (**a**) Top die, (**b**) Lower die, (**c**) Side die.

As shown in Figure 4, the wheel mold was constructed using Solidworks, which also created the top and lower molds, four side molds, a riser tube, and an insulation sleeve. The mold’s cooling system is shown in Figure 5 and Figure 6.

#### 2.2.2. Set Initial Conditions

**Figure 7:**
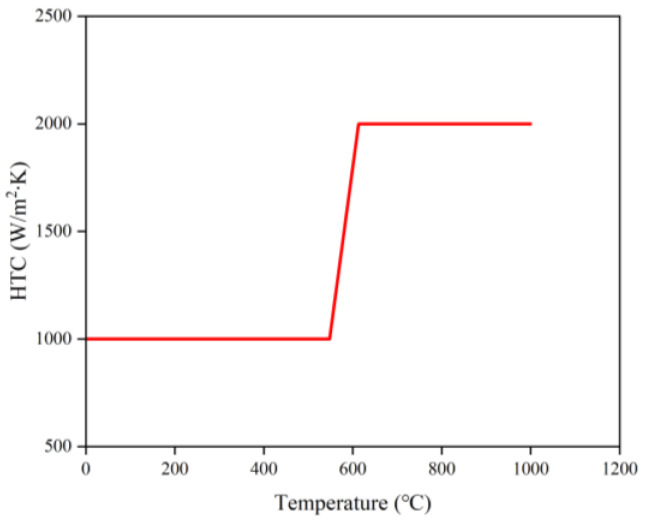
Heat transfer coefficient between metal fluid and mold.

Following the manufacturing practice, ceramic is used for the riser tube and H13 steel is chosen for the mold. The heat transmission coefficient between the mold and the surrounding atmosphere is 25 W/m^2^ K, and the working environment temperature is 25 °C. The heat transmission coefficient between the molds is set to EQUIV (means the two regions are continuous media, with a constant temperature distribution at the interface and a continuous velocity field) because they are made of the same material. Figure 7 illustrates how temperature [34] affects the heat transfer coefficient between the metal fluid and the mold.

The cooling process is very important to the solidification sequence of castings. The location of the cooling channel, the cooling intensity, and the opening and closing time of cooling all affect the solidification sequence of castings. In order to make the wheel conform to the principle of sequential solidification, the cooling process of the wheel should be optimized before the casting process optimization. Therefore, in the preliminary preparation work, the wheel hub was first simulated according to the original process of the factory, mainly for the optimization of its cooling process. Through several numerical simulations and test verification, the cooling process scheme was determined, as shown in Table 4, so that the wheel hub conforms to the sequential solidification under the original casting process.

As can be seen from the table, under some of the same cooling conditions, HTC is not the same, which is caused by considering the cooling intensity. The cooling intensity is related to the medium, flow rate, etc. The cooling intensity required for L5 and L7 locations is larger, so the HTC at this location is larger than that at other locations under the same conditions. Similarly, T1 is for cooling the riser tube. To improve production efficiency, a large cooling intensity is required, so its HTC is also larger than other locations under the same conditions.

#### 2.2.3. Collecting Response Data

According to the BBD test design, the commercial simulation software ProCAST was used to simulate the low-pressure casting process of large aluminum alloy 29 times, so as to obtain the defect volume fraction and the average secondary dendrite spacing of the spoke large plane under different process parameter combinations, as shown in Table 5.

Since the simulation results are uncertain, to improve the accuracy of the subsequent mathematical model as much as possible, it is necessary to retain more significant digits, even if it is not actually measurable. The accuracy of the mathematical model is the premise of its predictive ability.

### 2.3. Statistical Analysis and Optimization

#### 2.3.1. Building a Nonlinear Regression Model

The simulation output data, such as the defect volume fraction and average SDAS of the spoke surface, were gathered to establish the nonlinear input–output relationship with the process parameters. Following the model’s construction through regression analysis, its significance and predictive power were evaluated using analysis of variance (ANOVA) [35].

#### 2.3.2. Multi-Objective Optimization

In this work, we used the ISIGHT software along with the NSGA-II algorithm to optimize the process parameters and obtain the approximated globally optimal solution based on the mapping model and multi-objective solution, thus suggesting a more precise and efficient solution for the combination of process parameters [36].

### 2.4. Correction Optimization Program

Zhou et al. [36] optimized extrusion casting parameters based on the response surface, but did not consider that the change in response parameters would also affect the effect of the original cooling process. Therefore, this paper aims to correct the mold cooling process to make systemic optimization.

It is assumed that all other process parameters stay the same when determining the cooling process parameters used in this paper. Changes in the process parameters during parameter optimization may prevent the original cooling method from guaranteeing the hub’s sequential solidification. Therefore, it is important to confirm that the optimized process still adheres to the principle of sequential solidification under the cooling parameters and, if not, to make the necessary corrections.

### 2.5. Test Verification

Through methodical optimization of the process parameters, the obtained optimal combination of process parameters is validated in real production. Inspection of the large-size wheel molding quality and testing of the associated mechanical qualities serve as the primary means of confirming the viability of the enhanced process solution.

## 3. Results and Discussion

### 3.1. Defect Volume Fraction Analysis

Regression analysis using the defect volume fraction as a response indicator is given by:

(3) $$\begin{array}{cc} Y1=0.8202 & -\;0.2291X1-0.7834X2-0.5283X3-0.0099X4+0.1248X1X2+0.1267X1X3+0.1452X1X4\\ & +\;0.2030X2X3+0.0055X2X4-0.0165X3X4+0.2092{X1}^{2}+0.4022{X2}^{2}+0.3137{X3}^{2}\\ & +\;0.1115{X4}^{2} \end{array}$$

The fitted model was obtained, and the appropriateness of the model was assessed using an ANOVA, as shown in Table 6 and Table 7. The model’s extreme significance (*p*-value < 0.0001) and lack of significance (misfit) show that it can forecast the direction of the test factors’ influence on the response index.

An accurate model should have many coefficients of determination R_2_ that are bigger than 0.9 and closer to 1, but R_2_ also rises as more variables are added. As a result, the correlation is typically expressed by the modified R_2adj_. The coefficient of variation (C.V.) is less than 10%, which is within a reasonable range, and R_2_ and R_2adj_ in this model are more significant than 0.9, demonstrating the model’s high reliability [37].

**Figure 8:**
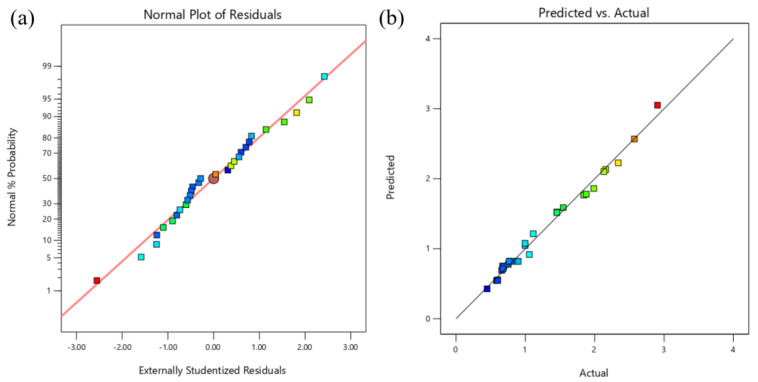
Response diagnostic chart: (**a**) Residual normal probability chart, (**b**) Predicted and actual distribution chart.

The anticipated response and actual values both follow a linear line distribution, as shown in Figure 8b, while the normal probability plot of the model residuals is presented in Figure 8a, whose residuals obey a normal distribution and follow a straight line.

**Figure 9:**
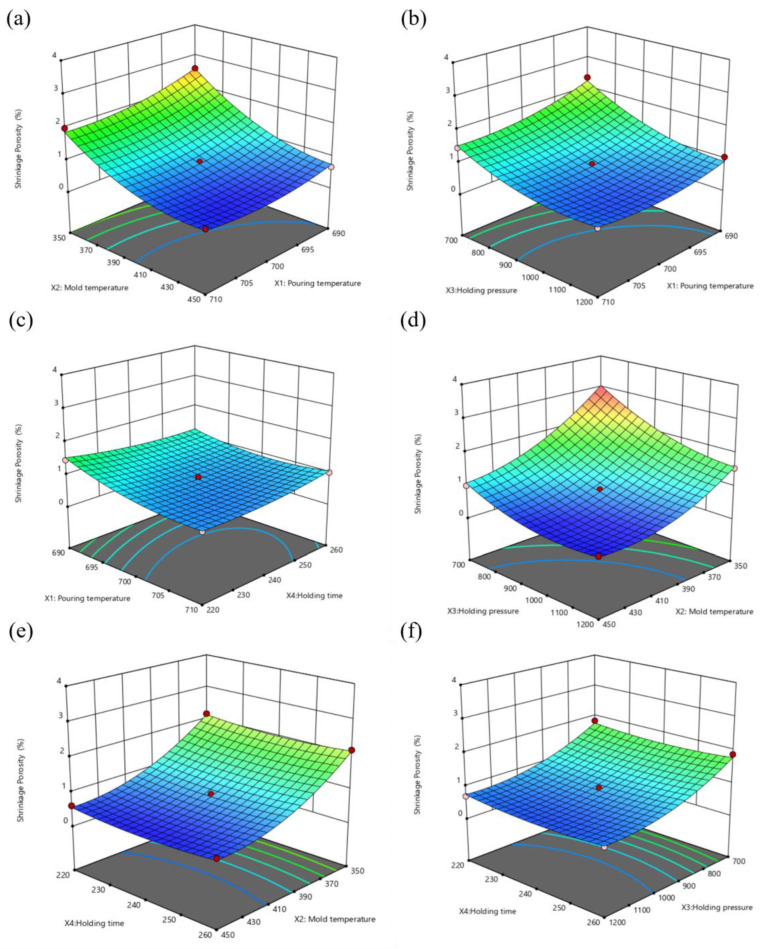
Defect volume fraction response surface plot: (**a**) X1 − X2, (**b**) X1 − X3, (**c**) X1 − X4, (**d**) X2 − X3, (**e**) X2 − X4, (**f**) X3 − X4.

The response surface model is shown in Figure 9. When X3 holding pressure and X4 holding duration are at the intermediate level, Figure 9a depicts the fluctuation of the reaction value Y1 defect volume fraction concerning X1 pouring temperature and X2 mold temperature. As can be seen from the figure, when X2 is between 400−450 °C, Y1 first decreases and then slightly increases with the increase of X1, while when X2 is between 350−400 °C, Y1 decreases with the increase of X1. Overall, Y1 decreases with the increase of X2. Moreover, within the selected level range, the influence rate of X2 is greater than that of X1, which indicates that increasing the mold temperature is an effective way to reduce the defect rate.

Figure 9b shows that Y1 decreases with the increase of X3, and when X3 is larger than 1050 mb, the change of Y1 tends to be stable. The obtained results are optimal when X1 is at a moderate level and X3 is greater than 1050 mb, which indicates that increasing the holding pressure can reduce the defect rate.

Both X2 and X3 have a substantial impact on Y1 as can be seen in Figure 9d, but when X2 is greater than 410 °C and X3 is greater than 1050 mb, the impact on Y1 is small, and the steepness of the curve demonstrates that X2 has a higher impact than X3. Similar to this, Figure 9c,e,f show that X4 has a negligible impact on Y1 and that Y1 shrinks slightly as X4 increases.

Through the analysis of the response surface of the volume fraction of defects, it can be concluded that the influence rate of mold temperature, holding pressure, pouring temperature, and holding time on the defects decrease in sequence within the set range. In the optimization of defects, increasing the mold temperature or increasing the holding pressure is the most effective way to reduce defects. In addition, appropriately increasing the pouring temperature is also beneficial to the reduction of defects. However, the pressure holding time has little influence on the defect, so the pressure holding time can be reduced to the lowest level in the selected range under the consideration of production efficiency.

### 3.2. Secondary Dendrite Spacing Analysis

The regression formula uses the average SDAS in the wide spoke plane as the response metric, and it is given by:

(4) $$\begin{array}{cc} Y2=50.24 & -\;0.3317X1+5.94X2+0.5883X3-0.4025X4-0.3125X1X2-0.6050X1X3+0.8225X1X4\\ & -\;0.2925X2X3-0.9825X2X4-0.4625X3X4+0.1828{X1}^{2}+0.9915{X2}^{2}+0.5152{X3}^{2}\\ & +\;0.0890{X4}^{2} \end{array}$$

Table 8 and Table 9 present the SDAS response model ANOVA. The model has strong predictive power, as evidenced by the *p*-value of <0.0001 and the insignificance of the out-of-fit term. With a C.V. of 1.12% and values for R^2^ and R^2^_adj_ that are close to 1, the model is considered to be reliable.

**Figure 10:**
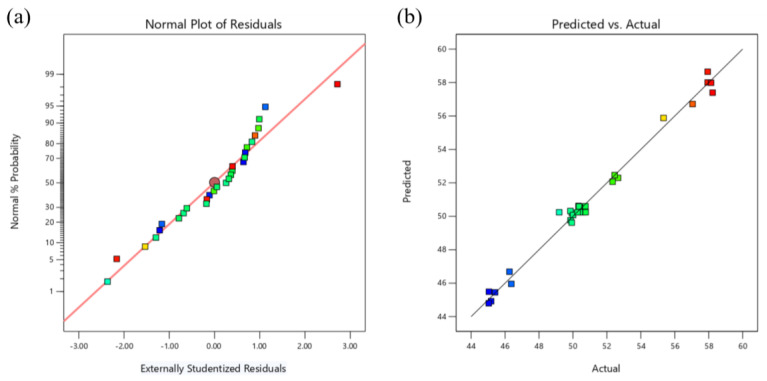
Response diagnostic chart: (**a**) Residual normal probability chart, (**b**) Predicted and actual distribution chart.

Figure 10a displays the normal probability plot of the model residuals, which exhibit a normal distribution and linear distribution, respectively. Figure 10b displays the anticipated response values and the actual response values, both of which exhibit a linear distribution.

**Figure 11:**
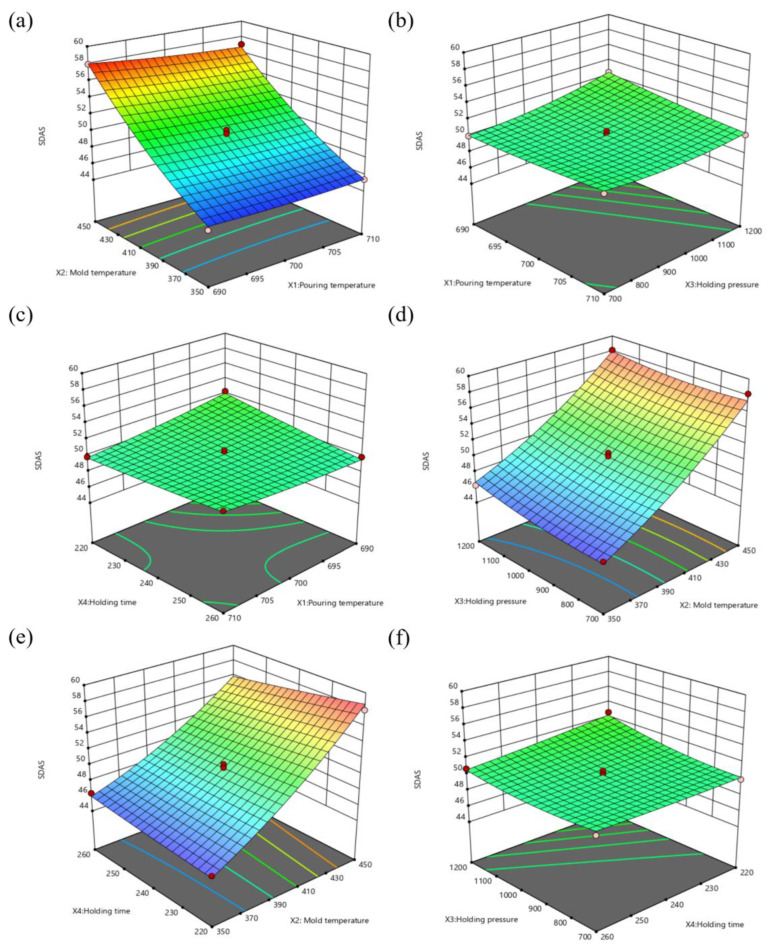
Spoke large plane average SDAS response surface plot: (**a**) X1 − X2, (**b**) X1 − X3, (**c**) X1 − X4, (**d**) X2 − X3, (**e**) X2 − X4, (**f**) X3 − X4.

In Figure 11, the response model is displayed. When observed in Figure 11a, Y2 decreases when X2 decreases; Y2 decreases as X1 increases when X2 is between 420−450 °C; and changes in X1 have little effect on Y2 when X2 is between 350−420 °C. Additionally, X2′s steepness is significantly greater than X1′s, demonstrating that mold temperature has a dominant effect on SDAS.

From Figure 11b, it is clear that when X1 is between 690−705 °C, Y2 grows with the increase of X3, and when X1 is greater than 705 °C, Y2 is little affected by the change in X3. When X3 is between 850−1200 mb, Y2 somewhat decreases with the increase of X1, and the opposite is true when X3 is less than 800 mb.

As can be seen in Figure 11c, when X1 is between 690−705 °C, Y2 decreases with the increase of X4. The reverse is true when X1 is greater than 705 °C. When X4 ranges from 220−240 s, Y2 decreases with the increase of X1. When X4 is in the 240−260 s range, the reverse is true. Moreover, the slope of X4 is slightly larger than that of X1; this shows that increasing the holding time is a great option to lower the SDAS when the pouring temperature is less than 705 °C.

It is evident from Figure 11d,e that the most important component in determining Y2 is X2. When X2 is less than 390 °C, Y2 increases with the increase of X3, and when X2 is greater than 390 °C, Y2 decreases with the increase of X4. This suggests that the holding pressure can be appropriately reduced at low mold temperatures, while SDAS can be reduced by prolonging the holding time at high mold temperatures.

According to Figure 11f, Y2 decreases with an increase in X4 when X3 is greater than 850 mb, and when X3 is between 700−850 mb, an increase in X4 has no discernible effect. In general, Y2 increases with the increase of X3, but when X4 is between 220−240 s, X3 has a larger growth rate.

Mold temperature is shown to be a key factor in SDAS by the response surface analysis. In this selection range, it has a significantly higher influence rate than holding pressure, holding time, and pouring temperature. To enhance the mechanical qualities of the wheel, the SDAS can be decreased by lowering the mold temperature and properly increasing the holding time.

### 3.3. Multi-Objective Optimization

#### 3.3.1. Building Mathematical Models

The commercial software Isight is a software for multi-disciplinary optimization design, which can realize multi-level intelligent optimization, multiple optimizations, and multi-level multi-disciplinary optimization methods. The genetic algorithm is chosen as the optimization strategy, because the genetic algorithm is iterative and has good convergence, and can expand the sampling space so that the optimization algorithm can find the global solution [38]. The most important evaluation metric for the low-pressure casting of large aluminum alloy wheels is the defect volume fraction. For low-pressure casting of large-size aluminum alloy wheels, the defect volume fraction is the most important evaluation index, so it is necessary to minimize the SDAS at the lowest possible defect volume. Multi-objective optimization was carried out according to the regression equation, and the weight coefficient was set as follows: defect volume fraction ω1 = 5, SDAS ω2 = 3, and the mathematical model was as follows:

(5) $$\begin{array}{c} \left\{\begin{array}{c} minf_{1}\left(X1,X2,X3,X4\right)=Y1\\ minf_{2}\left(X1,X2,X3,X4\right)=Y2 \end{array}\right.\\ 690\leq X1\leq 710;350\leq X2\leq 450;700\leq X3\leq 1200;220\leq X4\leq 260 \end{array}$$

**Figure 12:**
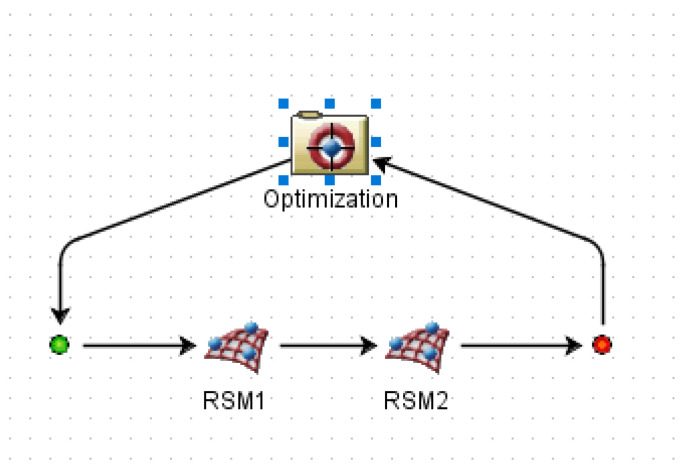
Optimization flow chart.

**Figure 13:**
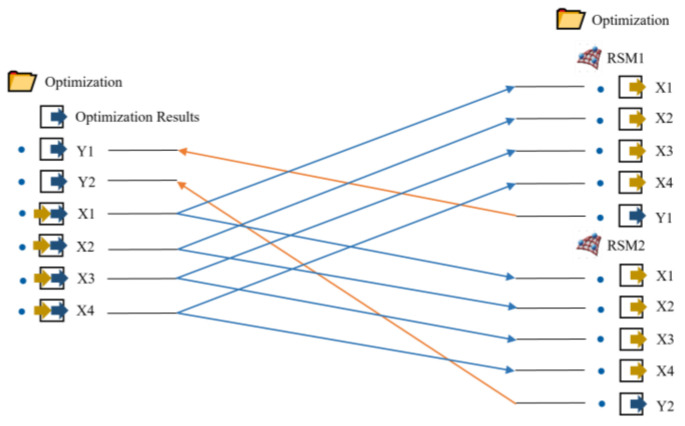
Variable mapping relationship chart.

The target variables are Y1 defect volume fraction and Y2 spoke large plane average SDAS, whereas the design factors are X1 pouring temperature, X2 mold temperature, X3 holding pressure, and X4 holding time. Figure 12 and Figure 13 show, respectively, the mapping relationships and optimization procedure. Table 10 displays the precise characteristics of the genetic algorithm program created using this mathematical model.

#### 3.3.2. Optimization Results and Validation

**Figure 14:**
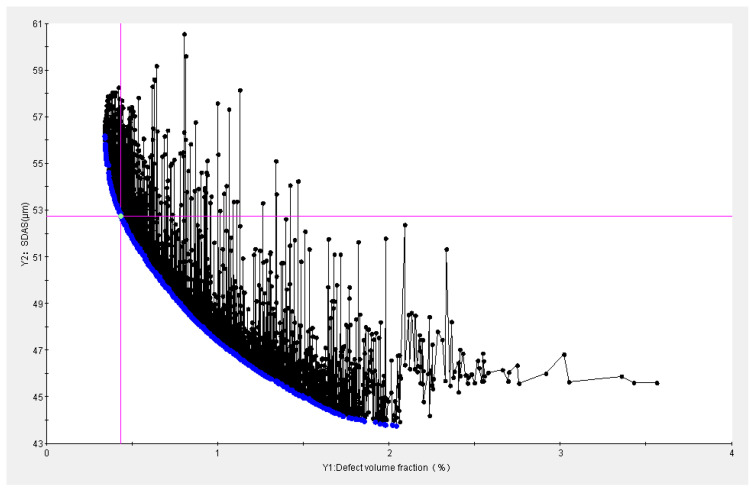
Distribution of Pareto solution scattering points.

Figure 14 displays the scatter distribution of the Pareto solution set for the optimization results, and Table 11 displays some of the solution sets.

The secondary dendrite spacing is inversely correlated with the faulty volume fraction. The casting and mold temperatures are essentially at the middle level, and the holding pressure and holding time are at the upper intermediate level, according to the Pareto-optimized solution set table. The distribution of the parameters is found to be consistent with the response surface analysis. According to the different weight coefficients, scheme No. 1 is the optimal solution. The approximate integer solution of the optimal solution—consisting of the optimized process parameters pouring temperature 703 °C, mold temperature 409 °C, holding pressure 1086 mb, and holding time 249 s—is chosen in consideration of the accuracy of production equipment.

**Figure 15:**
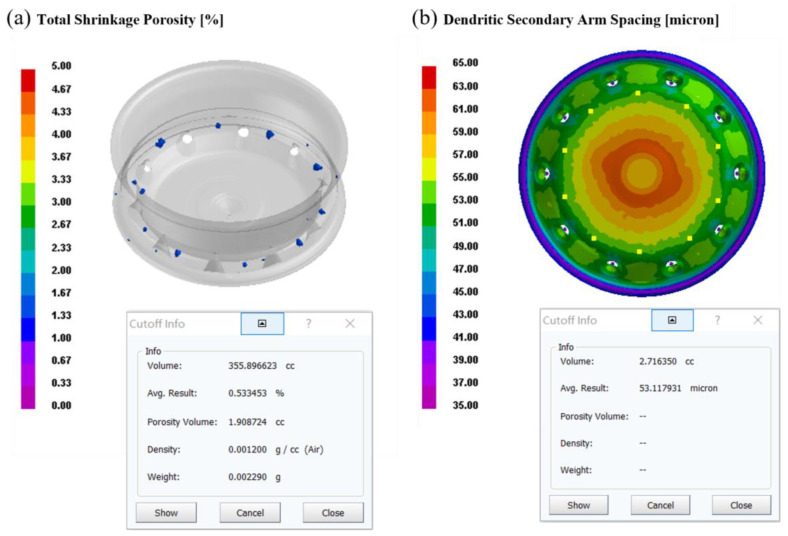
Optimized process simulation diagram: (**a**) Defect distribution map, (**b**) Secondary dendrite spacing map.

This set of process parameters was used to validate the numerical simulation, as illustrated in Figure 15. The average secondary dendrite spacing on the spoke surface is 53.12 µm, and the defect volume proportion is 0.533%. The simulation results are in line with the approximate model’s anticipated value, proving both the viability of the optimized process and the accuracy of the response model, which passed simulation verification.

### 3.4. Calibration of the Cooling Process

After multiple numerical simulations, the cooling process parameters used in this research were established and verified. There was no assurance that the wheels produced by the idealized set of process parameters would also adhere to sequential solidification during this cooling process due to variations in other process parameters. To accomplish systematic optimization, the original cooling process was adjusted.

**Figure 16:**
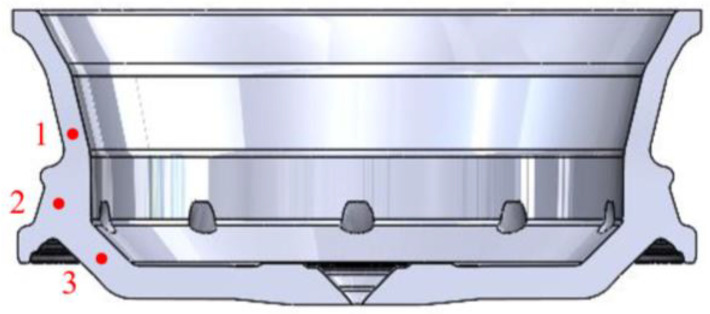
Solidification rate test point distribution map.

**Figure 17:**
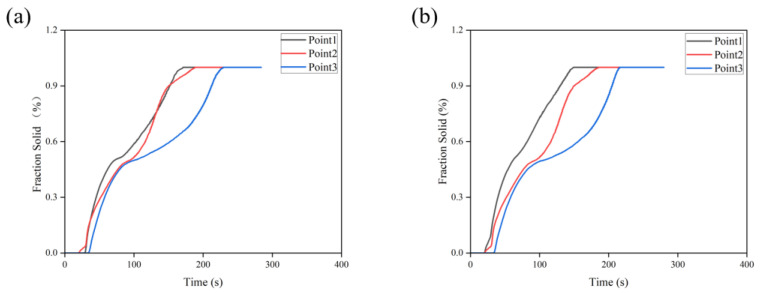
Variation curve of solidification fraction under different processes: (**a**) Solidification fraction curve under the original cooling process, (**b**) Solidification fraction curve after correction of the cooling process.

It can be seen from Figure 15a that the defects are concentrated at the junction of the spoked and rim. To make a better change and judge whether the optimized defect position is consistent with sequential solidification, three points are set in the simulation to measure the solidification rate, as shown in Figure 16. Figure 17 shows the change curve of the solidification fraction under the original cooling process. Ideally, the solidification fraction of No. 1 to No. 3 at the same time should be ω1 > ω2 > ω3. It is obvious from the figure that the curves overlap and do not conform to the sequence solidification. Therefore, the opening time of the cooling point at this position is adjusted to make it solidify sequentially. This position mainly corresponds to the thick part of the wheel hub, and the T4 and T5 cooling points of the top die are for the cooling of this position, so the two cooling points are selected to be fine-tuned. According to Figure 17a, the cooling point T5 should be switched on for 3 s in advance, and T4 should be delayed for 2 s, that is, the solidification of position 1 should be accelerated while the solidification of position 3 should be delayed. The corrected solidification fraction curve is shown in Figure 17b. The curves do not overlap or intersect, indicating that the region has been sequentially solidified, and the correction plan is feasible. The calibration cooling point process parameters are shown in Table 12.

The defect volume fraction decreased from 0.533% to 0.507%, and the secondary dendrite spacing remained unchanged, which further improved the casting quality. Compared with Zhou et al.’s [36] research, this paper realizes the systematic optimization of the large-size hub low-pressure casting process from the comprehensive optimization of process parameters to the correction of the cooling process.

## 4. Experimental Verification

### 4.1. X-ray Detection

**Figure 18:**
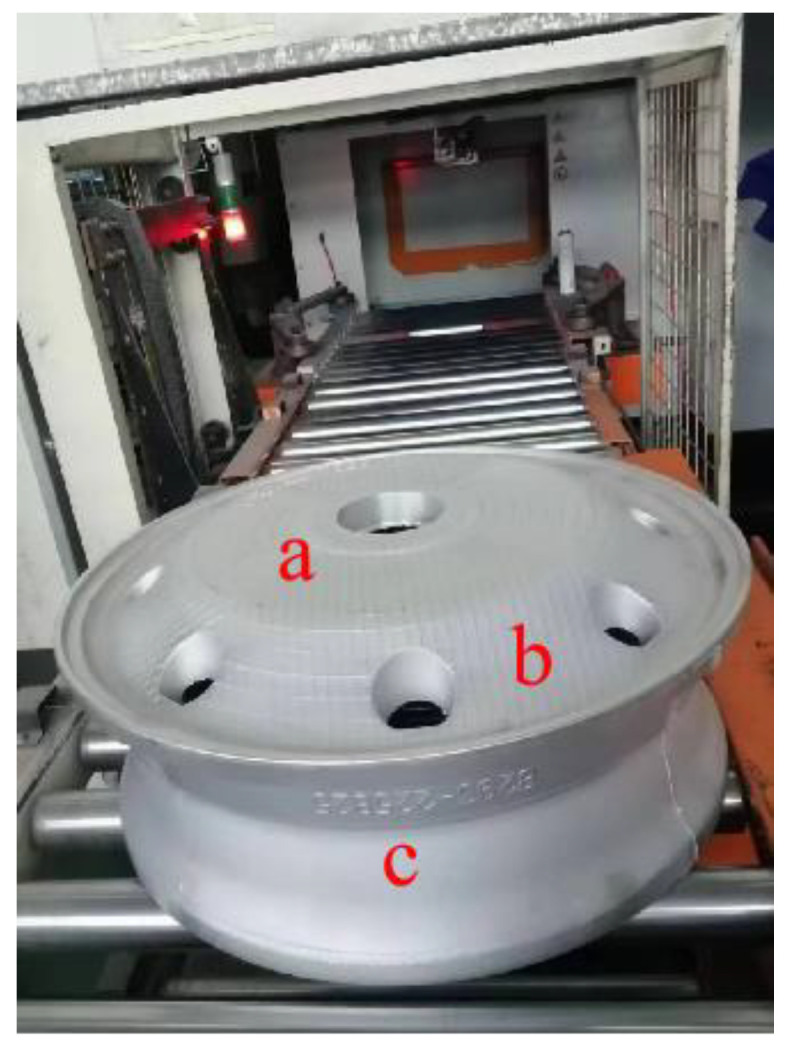
X-ray inspection location map.

**Figure 19:**
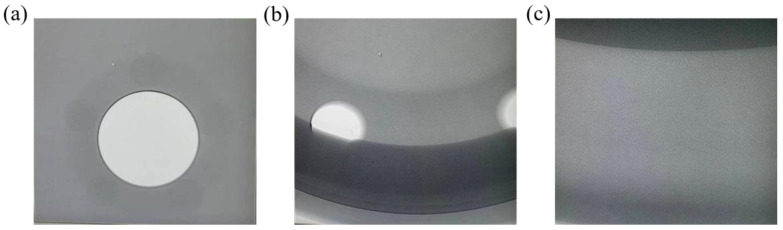
X-ray inspection diagram of different positions: (**a**) Spoke, (**b**) Intersection of spoke and rim, (**c**) Rim.

The wheel proto-type was produced utilizing the improved method. An X-ray was used to look for flaws inside, as can be shown in Figure 18. According to Figure 19, the spokes, rim intersection, and edge were all examined using X-rays. The maximum level of discontinuity detected by X-ray should conform to ASTM E155 [39]. The spoke surface of the wheel adopted a 1-level defect grade standard, and the junction between the spoke and the rim and the rim part adopted a 3-level defect grade standard. The system resolution met the requirements of GB/T23903, and JB/T7902 linear IQI was selected. The test results show that there was no defect of the corresponding level in the wheel hub, which proves that the wheel was made using this improved procedure and is of high quality.

The accuracy of X flaw detection is unable to detect defects below 1%. This means that the actual defect rate is below 1%, which is the same micro defect as the simulation result of 0.513%. This proves from the side that the error between the simulation and the actual is in a small range.

### 4.2. Mechanical Performance Analysis

**Figure 20:**
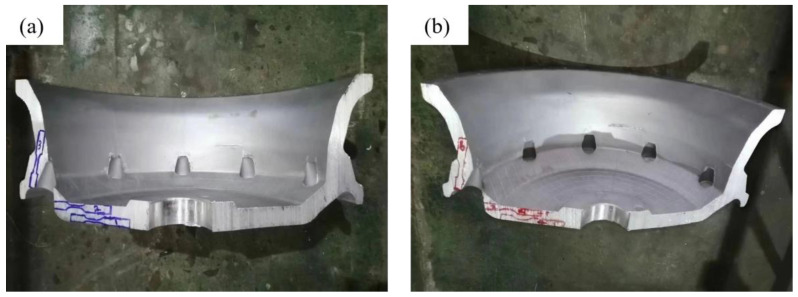
Sampling location diagram under different processes: (**a**) Original process hub sampling location map, (**b**) Optimized process sampling location map.

**Figure 21:**
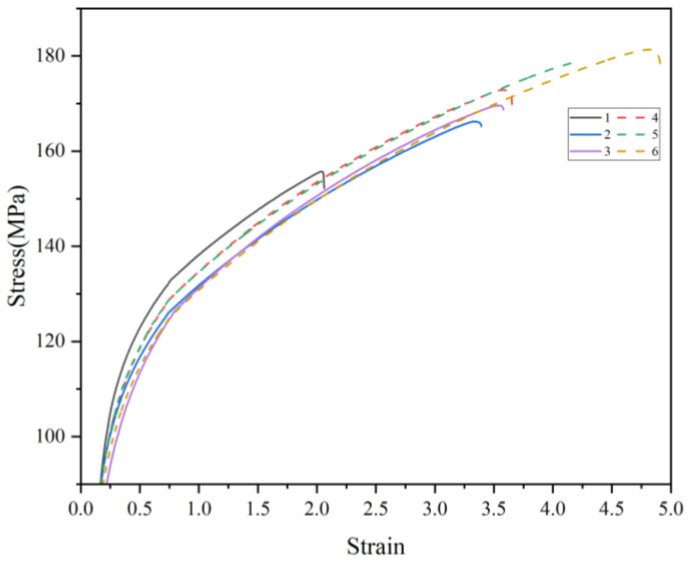
Comparison of the stress–strain curve.

**Figure 22:**
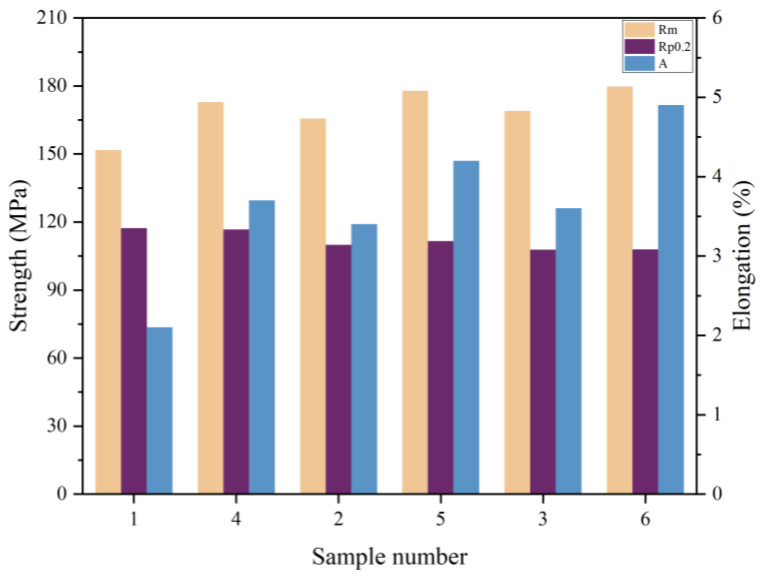
Comparative graph of mechanical performance tests.

The tensile strength, yield strength, and elongation of the as-cast condition wheel produced by the method were tested and compared to the characteristics of the as-cast condition wheel produced by the original production process to demonstrate the viability of the optimized process. The giant plane of the spokes is a concentrated part of the force. The sampling locations are primarily at the spoke surface because the mechanical qualities of this section must be high. The simulated defects are shown in Figure 20. According to the illustration, locations 4, 5, and 6 of the optimized process correspond to sample points 1, 2, and 3 of the original process, respectively. The test findings, which are displayed in Table 13 and Figure 21 and Figure 22, demonstrate that while the tensile strength and elongation are significantly enhanced, the yield strength at the same place does not change greatly, further demonstrating the viability of the optimized technique.

### 4.3. Metallographic Organization Analysis

**Figure 23:**
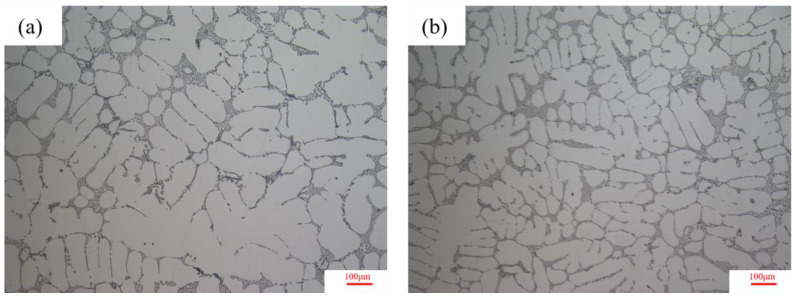
Local metallographic organization of the spoke surface under different processes: (**a**) Local metallographic view of the spoke surface of the original process wheel, (**b**) Local metallographic view of the spoke surface of the optimized process wheel.

To look inside the casting microstructure, sampling occurred at the spokes large plane, respectively on the original process and optimization process with location model and microstructure observation, as shown in Figure 23. It can be seen from the metallographic structure picture that there is no obvious defect in both of them, but it is found that the grain size is relatively small and the microstructure is relatively dense under the optimized process. The commercial particle size distribution statistics software, Nano measure, was used to measure its SDAS. Three photographs were used for each sample and twenty-five samples were taken for each photograph. The statistical results show that the SDAS of the original process is 48.1 ± 0.5 μm, and that of the optimized process is 37.9 ± 0.5 μm. This indicates that under the optimized process, the SDAS is further reduced, the grain is refined, and its metallographic structure is finer, so the mechanical properties are improved.

## 5. Conclusions

The following conclusions are reached after looking at a methodical strategy to optimize the low-pressure casting technique’s process parameters for large-size aluminum alloy wheels:

1. A simulation test software was performed based on RSM-BBD. Comprehensive research was conducted to determine the effects of pouring temperature, mold temperature, holding pressure, and holding time on the volume percentage of wheel faults and the average SDAS of the large plane of the wheel spokes;
2. The regression equation between the process parameters and the response target was created. The analysis of variance (ANOVA) revealed that the model is reliable and can be used to predict the defect volume % and SDAS;
3. The ISIGHT software’s NSGA-II genetic algorithm was used to multi-objectively optimize the regression model. The ideal process parameters were found using the Pareto solution set to be a pouring temperature of 703 °C, mold temperature of 409 °C, holding pressure of 1086 mb, and holding time of 249 s;
4. After optimizing the casting process, it was further verified by simulation whether it conformed to the principle of sequential solidification under the original cooling process. According to the analysis of the local solidification rate, it was found that the solidification curves crossed and did not achieve sequential solidification. Therefore, the corresponding cooling process parameters were adjusted according to the appearance time of the crossing node, that is, T5 starts cooling 3 s in advance and T4 starts cooling 2 s later. After adjustment, the solidification curves did not cross and the sequence solidification was realized. The process optimization was further improved to make it more systematic;
5. The wheel hub was manufactured by the optimized technology, and the X-ray test results showed that there were no X-ray defects in the casting. The mechanical properties and metallographic structure were analyzed by sampling at the large plane of the spoke. Compared with the original process, it is found that the mechanical properties of the wheel are improved under the optimized process. From the view of the metallographic structure, the optimized wheel hub structure is more tight, and its average SDAS is about 10 mm smaller than that of the original process. The feasibility of the optimized process is proved;
6. The mathematical model established in this paper is verified by the test, and it is found that the best process scheme predicted by the model is indeed better than the original process. Although there is a certain error between the simulated SDAS and the actual SDAS, it can still be used to qualitatively analyze and reflect the trend due to the uncertainty of both the simulated and measured results. This method provides a valuable idea for the process optimization of wheel manufacturers.

## Figures and Tables

**Table 1:** A356 material composition table.

**Element**	**Si**	**Fe**	**Cu**	**Mn**	**Mg**	**Ti**	**Sn**	**Ni**
Mass Fraction (%)	6.64	0.150	0.0018	0.0046	0.346	0.127	0.0100	0.0060
**Element**	**P**	**Zn**	**Pb**	**Sb**	**Sr**	**Ca**	**Cr**	**Al**
Mass Fraction (%)	0.0011	0.0101	0.0032	<0.0030	0.0126	0.00068	0.0035	93.19

**Table 2:** Experimental variables and levels.

Process Parameters	ID	Level 1	Level 2	Level 3
Pouring temperature (°C)	X1	690	700	710
Mold temperature (°C)	X2	350	400	450
Holding pressure (mb)	X3	700	950	1200
Holding time (s)	X4	220	240	260

**Table 3:** BBD experimental design scheme.

Run	Factor 1	Factor 2	Factor 3	Factor 4
	X1: Pouring Temperature (°C)	X2: Mold Temperature (°C)	X3: Holding Pressure (mb)	X4: Holding Time (s)
1st	700	450	1200	240
2nd	690	400	1200	240
3rd	710	450	950	240
4th	710	400	950	260
5th	710	400	700	240
6th	700	400	950	240
7th	710	400	1200	240
8th	710	400	950	220
9th	700	400	700	220
10th	700	450	950	220
11th	700	450	950	260
12th	710	350	950	240
13th	700	400	950	240
14th	700	400	700	260
15th	690	350	950	240
16th	690	450	950	240
17th	700	400	950	240
18th	700	450	700	240
19th	690	400	700	240
20th	700	400	1200	260
21st	700	400	950	240
22nd	700	350	700	240
23rd	700	350	950	260
24th	690	400	950	260
25th	700	350	950	220
26th	700	350	1200	240
27th	700	400	950	240
28th	700	400	1200	220
29th	690	400	950	220

**Table 4:** Opening and closing time of each cooling point and the value of HTC.

Location	Cooling Process	Open Time	Close Time	HTC(W/m^2^∙K)
L1	Air cooling	170	290	600
L2	Air cooling	160	290	600
L3	Air cooling	155	290	600
L4	Air cooling	140	260	600
L5	Air cooling	105	220	1500
L6	Water cooling	105	220	5000
L7	Air cooling	100	200	1500
T1	Water cooling	280	320	6000
T2	Air cooling	240	320	600
T3 (Close)	Air cooling	180	320	600
T4	Air cooling	125	160	600
T5	Air cooling	120	160	600
T6	Air cooling	40	240	600
A Channel	Air cooling	50	120	600
C Channel	Air cooling	80	160	600
S1	Air cooling	55	160	600
S2	Water cooling	80	160	5000

**Table 5:** Simulation-based estimates.

Run	Y1: Defect Volume (%)	Y2: Spoke Large Plane Average SDAS (µm)
1st	0.449	58.14 ± 1.37
2nd	1.057	52.46 ± 1.01
3rd	0.585	57.05 ± 0.83
4th	0.997	50.74 ± 1.24
5th	1.455	50.33 ± 1.57
6th	0.793	50.75 ± 0.96
7th	0.679	50.36 ± 1.44
8th	0.754	49.86 ± 1.25
9th	1.843	49.94 ± 1.13
10th	0.603	57.94 ± 1.03
11th	0.599	55.34 ± 0.87
12th	1.987	45.41 ± 1.36
13th	0.873	50.41 ± 1.15
14th	1.879	49.85 ± 1.16
15th	2.572	45.05 ± 1.44
16th	0.671	57.94 ± 1.03
17th	0.766	50.27 ± 0.98
18th	0.996	58.23 ± 1.25
19th	2.34	50.01 ± 1.13
20th	0.659	50.72 ± 1.01
21st	0.771	50.59 ± 1.32
22nd	2.906	45.18 ± 0.99
23rd	2.133	46.37 ± 1.24
24th	1.114	49.93 ± 1.51
25th	2.159	45.04 ± 1.25
26th	1.547	46.26 ± 1.12
27th	0.898	49.19 ± 1.06
28th	0.689	52.66 ± 1.26
29th	1.452	52.34 ± 0.87

**Table 6:** Defect volume fraction response model analysis of variance.

Source	Sum of Squares	df	Mean Square	F-Value	*p*-Value	
Model	13.22	14	0.9439	87.62	<0.0001	significant
X1	0.6298	1	0.6298	58.45	<0.0001	
X2	7.36	1	7.36	683.61	<0.0001	
X3	3.35	1	3.35	310.81	<0.0001	
X4	0.0012	1	0.0012	0.1095	0.7456	
X1X2	0.0623	1	0.0623	5.78	0.0306	
X1X3	0.0643	1	0.0643	5.96	0.0285	
X1X4	0.0844	1	0.0844	7.83	0.0142	
X2X3	0.1648	1	0.1648	15.30	0.0016	
X2X4	0.0001	1	0.0001	0.0112	0.9171	
X3X4	0.0011	1	0.0011	0.1011	0.7552	
X1^2^	0.2840	1	0.2840	26.36	0.0002	
X2^2^	1.05	1	1.05	97.41	<0.0001	
X3^2^	0.6385	1	0.6385	59.26	<0.0001	
X4^2^	0.0806	1	0.0806	7.48	0.0161	
Residual	0.1508	14	0.0108			
Lack of Fit	0.1359	10	0.0136	3.64	0.1124	not significant
Pure Error	0.0149	4	0.0037			
Cor Total	13.37	28				

**Table 7:** Summary of defect volume fraction model fitting.

Std.Dev	Mean	R^2^	Adjusted R^2^	C.V.%	Precision AP
0.1038	1.25	0.9887	0.9774	8.31	35.1420

**Table 8:** Analysis of variance for the secondary dendrite spacing response model.

Source	Sum of Squares	df	Mean Square	F-Value	*p*-Value	
Model	448.30	14	32.02	97.49	<0.0001	significant
X1	1.32	1	1.32	4.02	0.0647	
X2	424.00	1	424.00	1290.85	<0.0001	
X3	4.15	1	4.15	12.65	0.0032	
X4	1.94	1	1.94	5.92	0.0290	
X1X2	0.3906	1	0.3906	1.19	0.2939	
X1X3	1.46	1	1.46	4.46	0.0532	
X1X4	2.71	1	2.71	8.24	0.0123	
X2X3	0.3422	1	0.3422	1.04	0.3247	
X2X4	3.86	1	3.86	11.76	0.0041	
X3X4	0.8556	1	0.8556	2.60	0.1288	
X1^2^	0.2166	1	0.2166	0.6595	0.4303	
X2^2^	6.38	1	6.38	19.41	0.0006	
X3^2^	1.78	1	1.72	5.24	0.0381	
X4^2^	0.0514	1	0.0514	0.1564	0.6984	
Residual	4.60	14	0.3285			
Lack of Fit	3.08	10	0.3084	0.8142	0.6411	not significant
Pure Error	1.51	4	0.3787			
Cor Total	452.90	28				

**Table 9:** Summary of secondary dendrite spacing model fits.

Std.Dev	Mean	R^2^	Adjusted R^2^	C.V.%	Precision AP
0.5731	50.98	0.9898	0.9556	1.12	33.6096

**Table 10:** Genetic algorithm parameter settings.

Population Size	Number of Generations	Crossover Probability	Crossover Distribution Index	Mutation Distribution Index
200	20	0.9	10.0	20.0

**Table 11:** Part Pareto-optimized solution set table.

No.	X1 (°C)	X2 (°C)	X3 (mb)	X4 (s)	Y1 (%)	Y2 (µm)	Comment
1	703.18	408.96	1085.8	248.21	0.456	52.42	Optimal solution
2	702.86	421.52	1102.3	243.05	0.500	53.40	
3	709.97	371.96	1047.8	220.41	1.126	46.64	
4	703.54	409.21	1092.7	242.41	0.496	51.80	
5	707.99	397.21	1068.3	228.02	0.654	50.07	
6	702.53	415.28	1115.2	244.11	0.441	53.64	
7	709.72	384.97	1054.3	221.21	0.874	48.23	
8	702.53	405.89	1082.4	241.73	0.533	51.41	

**Table 12:** Cooling process calibration table.

Location	Cooling Process	Open Time (s)	Close Time (s)	HTC (W/m^2^∙K)
T4	Air cooling	125→127	160	600
T5	Air cooling	120→117	160	600

**Table 13:** Comparison table of different process properties.

Original Process				Optimized Process			
	Rm	Rp 0.2	A		Rm	Rp 0.2	A
No.	≥MPa	≥MPa	≥%	No	≥MPa	≥MPa	≥%
1	151.7	111.3	2.1	4	172.9	111.7	3.7
2	165.6	109.9	3.4	5	177.9	111.6	4.2
3	168.9	107.8	3.6	6	179.8	107.9	4.9

## Data Availability

Data is contained within the article.
